# A monoclinic polymorph of 4-(2*H*-1,3-benzodioxol-5-yl)-1-(4-methyl­phen­yl)-1*H*-pyrazol-5-amine

**DOI:** 10.1107/S2056989015016023

**Published:** 2015-09-12

**Authors:** Mukesh M. Jotani, Nilesh N. Gajera, Mukesh C. Patel, Herman H. Y. Sung, Edward R. T. Tiekink

**Affiliations:** a Department of Physics, Bhavan’s Sheth R. A. College of Science, Ahmedabad, Gujarat 380 001, India; b P. S. Science and H. D. Patel Arts College, S. V. Campus, Kadi, Gujarat 382 715, India; c Department of Chemistry, The Hong Kong University of Science and Technology, Clear Water Bay, Kowloon, Hong Kong, People’s Republic of China; d Department of Chemistry, University of Malaya, 50603 Kuala Lumpur, Malaysia; e Centre for Chemical Crystallography and Faculty of Science and Technology, Sunway University, 47500 Bandar Sunway, Selangor Darul Ehsan, Malaysia

**Keywords:** crystal structure, amine, polymorph, conformation, Hirshfeld surface

## Abstract

The title compound, C_17_H_15_N_3_O_2_, is a monoclinic polymorph (*P*2_1_/*c* with *Z*′ = 1) of the previously reported triclinic (*P*-1 with *Z*′ = 2) form [Gajera *et al.* (2013 ▸). *Acta Cryst.* E**69**, o736–o737]. The mol­ecule in the monoclinic polymorph features a central pyrazolyl ring with an N-bound *p*-tolyl group and a C-bound 1,3-benzodioxolyl fused-ring system on either side of the C atom bearing the amino group. The dihedral angles between the central ring and the N- and C-bound rings are 50.06 (5) and 27.27 (5)°, respectively. The angle between the pendent rings is 77.31 (4)°, indicating the mol­ecule has a twisted conformation. The five-membered dioxolyl ring has an envelope conformation with the methyl­ene C atom being the flap. The relative disposition of the amino and dioxolyl substituents is *syn*. One of the independent mol­ecules in the triclinic form has a similar *syn* disposition but the other has an *anti* arrangement of these substituents. In the crystal structure of the monoclinic form, mol­ecules assemble into supra­molecular helical chains *via* amino–pyrazolyl N—H⋯N hydrogen bonds. These are linked into layers *via* C—H⋯π inter­actions, and layers stack along the *a* axis with no specific inter­actions between them.

## Abstract 2

A second polymorph (monoclinic with *Z*′ = 1) of the title compound is reported in which the conformation resembles one of the independent mol­ecules of the original triclinic polymorph (*Z*′ = 2).

## Chemical context   

It is the broad range of biological activities, such as anti-depressant, anti-anxiety, anti-fungal, anti-bacterial, anti-diabetic, anti-cancer, *etc*. (Tanitame *et al.*, 2004 ▸; Chimenti *et al.*, 2006 ▸; Ding *et al.*,2009 ▸; Shen *et al.*, 2011 ▸; Deng *et al.*, 2012 ▸), that continues to inspire inter­est in compounds containing the amino-substituted pyrazole unit. It was in this context that the crystal structure of 4-(2*H*-1,3-benzodioxol-5-yl)-1-(4-methyl­phen­yl)-1*H*-pyrazol-5-amine (I) was originally determined (Gajera *et al.*, 2013 ▸). Subsequently, during scale up, crystals of the monoclinic form were isolated from recrystallization of (I) from ethyl acetate, the same solvent system that afforded the original triclinic polymorph. Herein, the crystal and mol­ecular structures of the monoclinic form of (I), hereafter (mI), are described and compared with the triclinic polymorph, (tI).
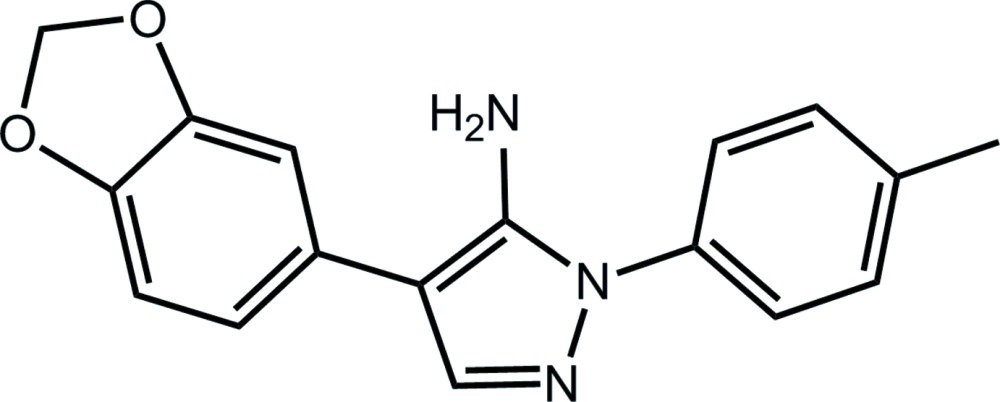


## Structural commentary   

**Figure 1:**
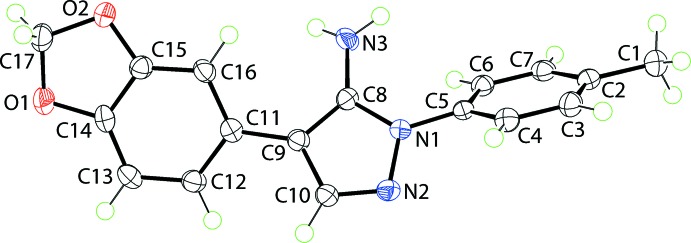
The mol­ecular structure of the mol­ecule found in the monoclinic polymorph showing the atom-labelling scheme and displacement ellipsoids at the 70% probability level.

The mol­ecule in (mI), Fig. 1 ▸, comprises a central and almost planar pyrazolyl ring (r.m.s. deviation of the five atoms = 0.0043 Å) flanked by an N-bound *p*-tolyl group and a C-bound 1,3-benzodioxolyl fused ring system. In the latter, the five-membered dioxolyl ring adopts an envelope conformation with the methyl­ene-C17 atom being the flap; the C17 atom lies 0.318 (2) Å out of the least-squares plane defined by the O1, O2, C14 and C15 atoms (r.m.s. deviation = 0.0005 Å). The dihedral angles between the central ring and the N- and C-bound six-membered rings are 50.06 (5) and 27.27 (5)°, respectively. The dihedral angle between the six-membered rings is 77.31 (4)°, indicating an overall twisted arrangement. In general terms, the relative disposition of the amino and dioxolyl substituents may be described as being *syn*.

**Figure 2:**
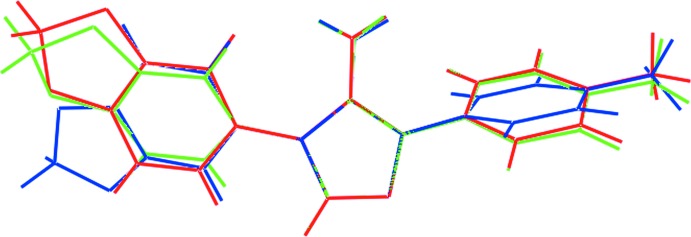
Overlay diagram of the title compound, (mI), red image, with the two independent mol­ecules in (tI), green (mol­ecule *a*) and blue (*b*) images. The mol­ecules have been overlapped so that the central pyrazolyl rings are coincident.

While (mI) crystallizes with *Z*′ = 1, the triclinic polymorph, (tI), crystallizes with *Z*′ = 2 (Gajera *et al.*, 2013 ▸). In the latter, the mol­ecules have quite different conformations. In one of the independent mol­ecules, the amino and dioxolyl substit­uents are *syn*, as for (mI), and in the other these substituents are *anti*. These differences in mol­ecular conformations are highlighted in Fig. 2 ▸. The *syn*/*anti* distinction is quite clear from this overlap diagram where the dioxolyl ring obviously occupies a different position in the second independent mol­ecule of (tI, blue image). Also evident from Fig. 2 ▸ are variations in the relative dispositions of six-membered rings. These variations are qu­anti­fied in Table 1 ▸.

## PXRD study   

In order to ascertain the nature of the crystalline residue isolated from recrystallization of (I) from ethyl acetate solution, a powder X-ray diffraction (PXRD) experiment was performed on a PANalytical Empyrean XRD system with Cu Kα1 radiation (λ = 1.54056 Å) in the 2θ range of 5 to 50° with a step size of 0.026°. The pattern was analyzed with *X’Pert HighScore Plus* (PANalytical, 2009 ▸). This analysis indicated that the ratio of (mI) to (tI) in the overall sample was 49.1:50.9. This distribution suggests that effectively in the sample there is a 3:1 ratio of mol­ecules with a *syn* disposition of the amino and dioxolyl substituents to those with a *trans* arrangement.

## Supra­molecular features   

**Figure 3:**
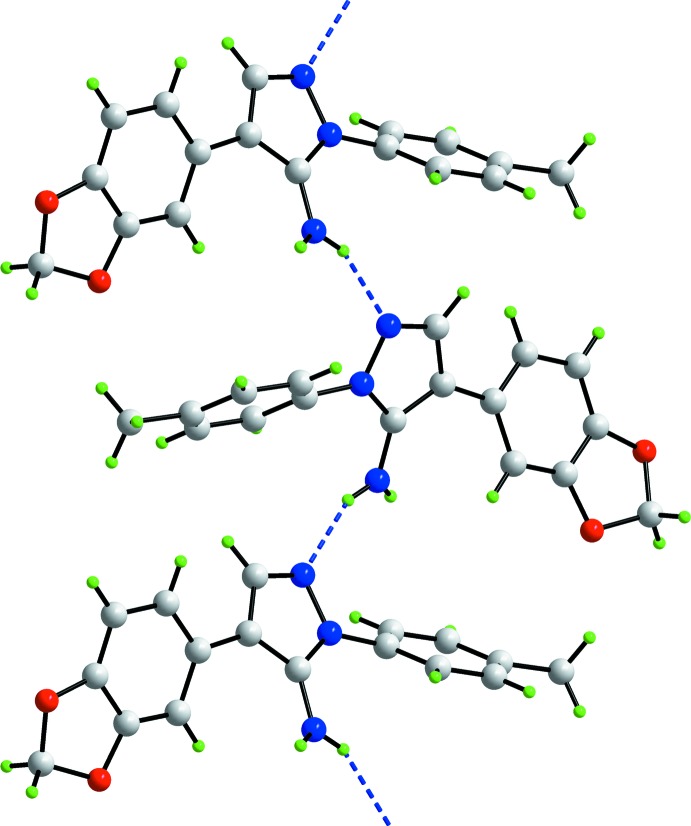
A view of a supra­molecular helical chain aligned along the *b* axis and mediated by amino–pyrazolyl N—H⋯N hydrogen bonds shown as blue dashed lines.

**Figure 4:**
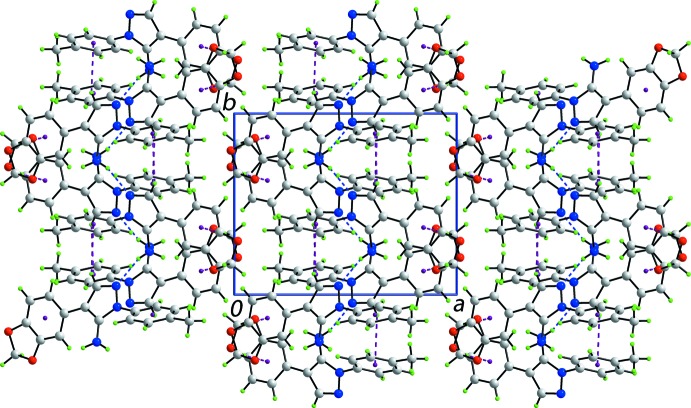
Unit-cell contents shown in projection down the *c* axis. The N—H⋯N and C—H⋯π inter­actions are shown as blue and purple dashed lines, respectively.

The most notable feature of the crystal packing in (mI) is the formation of supra­molecular helical chains aligned along the *b* axis and mediated by amino–pyrazolyl N—H⋯N hydrogen bonds, Fig. 3 ▸ and Table 2 ▸. The chains are consolidated into layers in the *bc* plane by pyrazol­yl–tolyl C10—H⋯π and methyl­ene–benzo-C_6_ C17—H⋯π inter­actions, Table 2 ▸. The layers inter-digitate along the *a* axis whereby the dioxolyl rings face each other, Fig. 4 ▸. The C—H⋯O inter­actions are at distances beyond the standard criteria (Spek, 2009 ▸). In the packing scheme just described, no specific role is found for the second amino-H2*N* atom. To a first approximation, the mode of association between mol­ecules in (tI) is similar in that supra­molecular chains are formed. These comprise alternating independent mol­ecules *a* and *b* that are connected by amino–pyrazolyl N—H⋯N hydrogen bonds. The difference is that in (tI), the chains have a zigzag topology. Chains in (tI) are connected by C—H⋯O and C—H⋯π inter­actions.

## Analysis of the Hirshfeld surfaces   

**Figure 5:**
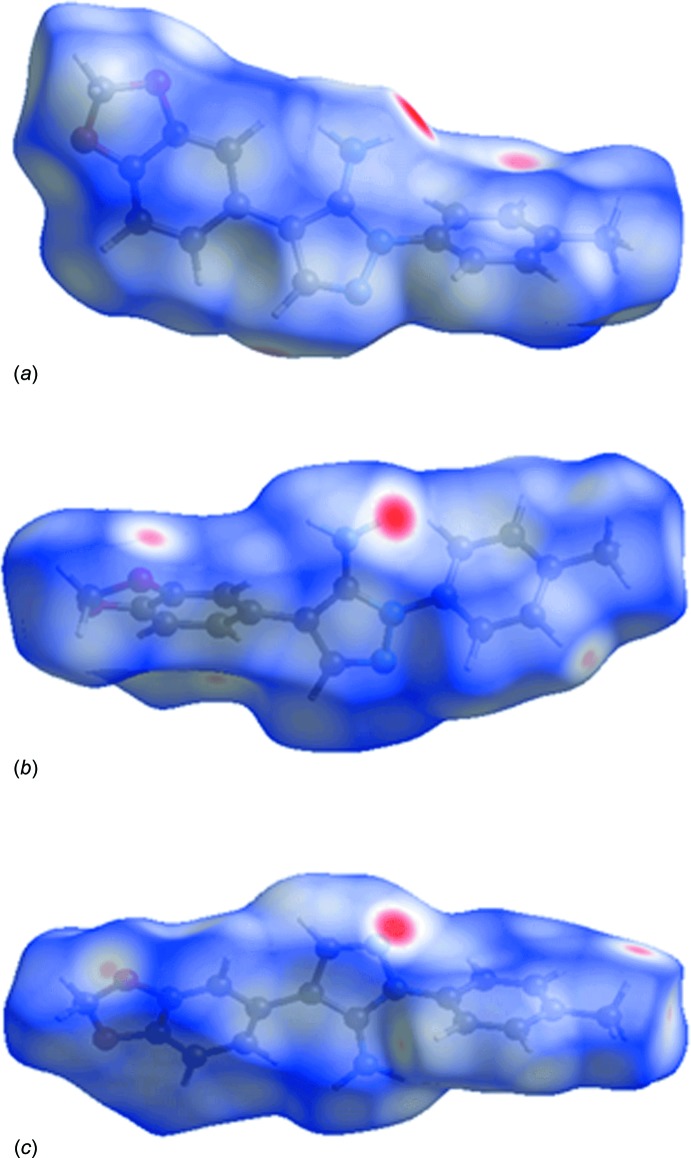
Views of the Hirshfeld surfaces for (*a*) (mI), (*b*) (tI) – mol­ecule *a*, and (*c*) (tI) – mol­ecule *b*.

**Figure 6:**
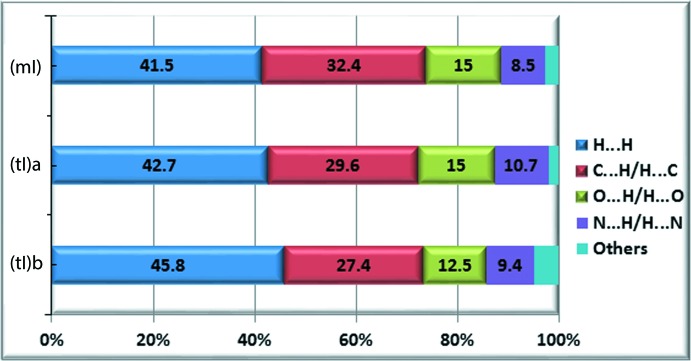
Relative contributions of various inter­molecular contacts to the Hirshfeld surface area in (*a*) mI, and of (tI) mol­ecules (*b*) *a* and (*c*) *b*.

In order to investigate further the nature of the crystal packing in (mI) and (tI), an analysis of the Hirshfeld surfaces (Spackman & Jayatilaka, 2009 ▸) was undertaken employing *CrystalExplorer* (Wolff *et al.*, 2012 ▸). The Hirshfeld surfaces were mapped over *d*_norm_ for each of the three mol­ecules, Fig. 5 ▸. The points of contact corresponding to the amino–pyrazolyl N—H⋯N hydrogen bonds are recognized easily by deep-red depressions on the Hirshfeld surfaces of all three mol­ecules. The C—H⋯π inter­actions in (mI) are indicated by both diminutive spots and light-red regions on the surface. These are also apparent in (tI) with additional features arising from the C—H⋯O contacts, Fig. 5 ▸. The fingerprint plots (Rohl *et al.*, 2008 ▸) were also calculated and enabled a delineation of the relative contribution of the different inter­molecular contacts to the respective crystal structures. These contributions are illustrated graphically in Fig. 6 ▸. Despite the different modes of association between the respective mol­ecules, to a first approximation the relative contributions to the surfaces are similar.

## Database survey   

A search of the Cambridge Structural Database (Groom & Allen, 2014 ▸), revealed there are no direct analogues of (I), *i.e*. 1,3 N- and C-disubstituted species. There are four examples of 1,3,4 tris­ubstituted analogues (Abu Thaher *et al.*, 2012 ▸; and references therein).

## Synthesis and crystallization   

The title compound was synthesized according to the same synthetic process as described in the original report (Gajera *et al.*, 2013 ▸). Single crystals suitable for X-ray measurements in the form of light-brown prisms were obtained from its ethyl acetate solution at room temperature.

## Refinement   

Crystal data, data collection and structure refinement details are summarized in Table 3 ▸. Carbon-bound H-atoms were placed in calculated positions (C—H = 0.95–0.99 Å) and were included in the refinement in the riding model approximation, with *U*_iso_(H) set to 1.2–1.5*U*_eq_(C). The N-bound H atoms were located in a difference Fourier map but were refined with a distance restraint of N—H = 0.88±0.01 Å, and with *U*_iso_(H) set to 1.2*U*_eq_(N).

## Supplementary Material

Crystal structure: contains datablock(s) I, global. DOI: 10.1107/S2056989015016023/hb7490sup1.cif

Structure factors: contains datablock(s) I. DOI: 10.1107/S2056989015016023/hb7490Isup2.hkl

Supporting information file. DOI: 10.1107/S2056989015016023/hb7490Isup3.cml

CCDC reference: 1420783

Additional supporting information:  crystallographic information; 3D view; checkCIF report

## Figures and Tables

**Table 1:** Dihedral angle () data for the three independent molecules in (mI) and (tI)

Structure	pyrazolyl/*p*-tolyl	pyrazolyl/benzo-C_6_	*p*-tolyl/benzo-C_6_
(mI)	50.06(5)	27.27(5)	77.31(4)
(tI), molecule *a*	49.08(9)	47.18(7)	85.22(8)
(tI), molecule *b*	68.22(9)	31.67(8)	80.63(8)

**Table 2:** Hydrogen-bond geometry (, ) *Cg*1 and *Cg*2 are the centroids of the C2C7 and C11C16 rings, respectively.

*D*H*A*	*D*H	H*A*	*D* *A*	*D*H*A*
N3H1*N*N2^i^	0.88(2)	2.16(2)	2.9981(16)	159(1)
C10H10*Cg*1^ii^	0.95	2.97	3.6753(14)	133
C17H17*B* *Cg*2^iii^	0.99	2.66	3.6334(15)	169

Symmetry codes: (i) 
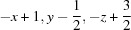
; (ii) 
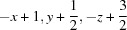
; (iii) 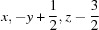.

**Table 3:** Experimental details

Crystal data	
Chemical formula	C_17_H_15_N_3_O_2_
*M*_r_	293.32
Crystal system, space group	Monoclinic, *P*2_1_/*c*
Temperature (K)	100
*a*, *b*, *c* ()	13.9652(3), 10.6898(2), 9.8459(2)
()	109.844(2)
*V* (^3^)	1382.57(5)
*Z*	4
Radiation type	Cu *K*
(mm^1^)	0.77
Crystal size (mm)	0.35 0.25 0.15

Data collection	
Diffractometer	Agilent SuperNova Dual diffractometer with an Atlas detector
Absorption correction	Multi-scan (*CrysAlis PRO*; Agilent, 2014 ▸)
*T*_min_, *T*_max_	0.989, 1.000
No. of measured, independent and observed [*I* > 2(*I*)] reflections	4379, 2582, 2289
*R*_int_	0.013
(sin /)_max_ (^1^)	0.609

Refinement	
*R*[*F* ^2^ > 2(*F* ^2^)], *wR*(*F* ^2^), *S*	0.036, 0.096, 1.03
No. of reflections	2582
No. of parameters	206
No. of restraints	2
H-atom treatment	H atoms treated by a mixture of independent and constrained refinement
_max_, _min_ (e ^3^)	0.19, 0.27

Computer programs: *CrysAlis PRO* (Agilent, 2014 ▸), *SHELXL97* (Sheldrick, 2008 ▸), *SHELXL2014* (Sheldrick, 2015 ▸), *ORTEP-3 for Windows* (Farrugia, 2012 ▸), *QMol* (Gans Shalloway, 2001 ▸), *DIAMOND* (Brandenburg, 2006 ▸) and *publCIF* (Westrip, 2010 ▸).

## supplementary crystallographic information

### Crystal data

**Table:**

C_17_H_15_N_3_O_2_	*F*(000) = 616
*M**_r_* = 293.32	*D*_x_ = 1.409 Mg m^−^^3^
Monoclinic, *P*2_1_/*c*	Cu *K*α radiation, λ = 1.54184 Å
*a* = 13.9652 (3) Å	Cell parameters from 2900 reflections
*b* = 10.6898 (2) Å	θ = 5.3–75.6°
*c* = 9.8459 (2) Å	µ = 0.77 mm^−^^1^
β = 109.844 (2)°	*T* = 100 K
*V* = 1382.57 (5) Å^3^	Prism, light-brown
*Z* = 4	0.35 × 0.25 × 0.15 mm

### Data collection

**Table:**

Agilent SuperNova Dual diffractometer with an Atlas detector	2582 independent reflections
Radiation source: SuperNova (Cu) X-ray Source	2289 reflections with *I* > 2σ(*I*)
Mirror monochromator	*R*_int_ = 0.013
ω scans	θ_max_ = 70.0°, θ_min_ = 5.3°
Absorption correction: multi-scan (*CrysAlis PRO*; Agilent, 2014)	*h* = −16→12
*T*_min_ = 0.989, *T*_max_ = 1.000	*k* = −12→12
4379 measured reflections	*l* = −11→11

### Refinement

**Table:**

Refinement on *F*^2^	2 restraints
Least-squares matrix: full	Hydrogen site location: mixed
*R*[*F*^2^ > 2σ(*F*^2^)] = 0.036	H atoms treated by a mixture of independent and constrained refinement
*wR*(*F*^2^) = 0.096	*w* = 1/[σ^2^(*F*_o_^2^) + (0.0527*P*)^2^ + 0.5203*P*] where *P* = (*F*_o_^2^ + 2*F*_c_^2^)/3
*S* = 1.03	(Δ/σ)_max_ < 0.001
2582 reflections	Δρ_max_ = 0.19 e Å^−^^3^
206 parameters	Δρ_min_ = −0.27 e Å^−^^3^

### Special details

**Table:**

Geometry. All e.s.d.'s (except the e.s.d. in the dihedral angle between two l.s. planes) are estimated using the full covariance matrix. The cell e.s.d.'s are taken into account individually in the estimation of e.s.d.'s in distances, angles and torsion angles; correlations between e.s.d.'s in cell parameters are only used when they are defined by crystal symmetry. An approximate (isotropic) treatment of cell e.s.d.'s is used for estimating e.s.d.'s involving l.s. planes.

### Fractional atomic coordinates and isotropic or equivalent isotropic displacement parameters (Å^2^)

**Table:**

	*x*	*y*	*z*	*U*_iso_*/*U*_eq_	
O1	−0.01538 (7)	0.79537 (9)	0.05812 (10)	0.0229 (2)	
O2	0.09248 (7)	0.63677 (9)	0.17958 (10)	0.0229 (2)	
N1	0.47346 (8)	0.93081 (10)	0.71284 (11)	0.0146 (2)	
N2	0.46867 (8)	1.04737 (10)	0.65038 (11)	0.0168 (2)	
N3	0.38155 (8)	0.73744 (11)	0.67648 (13)	0.0229 (3)	
H1N	0.4302 (10)	0.6978 (15)	0.7442 (15)	0.028*	
H2N	0.3203 (8)	0.7054 (15)	0.6459 (17)	0.028*	
C1	0.79815 (10)	0.85389 (13)	1.24901 (15)	0.0226 (3)	
H1A	0.7964	0.9221	1.3147	0.034*	
H1B	0.8637	0.8547	1.2330	0.034*	
H1C	0.7896	0.7737	1.2917	0.034*	
C2	0.71312 (10)	0.87116 (12)	1.10672 (14)	0.0178 (3)	
C3	0.73229 (9)	0.91569 (12)	0.98529 (14)	0.0188 (3)	
H3	0.8002	0.9347	0.9919	0.023*	
C4	0.65366 (9)	0.93269 (12)	0.85479 (14)	0.0173 (3)	
H4	0.6679	0.9627	0.7728	0.021*	
C5	0.55400 (9)	0.90549 (11)	0.84473 (13)	0.0146 (3)	
C6	0.53312 (9)	0.86113 (11)	0.96412 (13)	0.0161 (3)	
H6	0.4651	0.8427	0.9574	0.019*	
C7	0.61276 (10)	0.84398 (12)	1.09371 (14)	0.0174 (3)	
H7	0.5984	0.8130	1.1752	0.021*	
C8	0.39184 (9)	0.85823 (12)	0.63961 (13)	0.0149 (3)	
C9	0.33011 (9)	0.93033 (12)	0.52569 (13)	0.0154 (3)	
C10	0.38242 (10)	1.04516 (12)	0.54044 (13)	0.0170 (3)	
H10	0.3579	1.1141	0.4773	0.020*	
C11	0.23592 (9)	0.89631 (12)	0.40828 (13)	0.0157 (3)	
C12	0.16893 (9)	0.99103 (12)	0.33479 (14)	0.0180 (3)	
H12	0.1828	1.0747	0.3683	0.022*	
C13	0.08223 (10)	0.96750 (13)	0.21382 (14)	0.0199 (3)	
H13	0.0379	1.0328	0.1646	0.024*	
C14	0.06491 (9)	0.84515 (13)	0.17031 (13)	0.0177 (3)	
C15	0.12917 (9)	0.75009 (12)	0.24281 (14)	0.0170 (3)	
C16	0.21486 (9)	0.77116 (12)	0.36133 (14)	0.0169 (3)	
H16	0.2580	0.7044	0.4095	0.020*	
C17	0.01608 (10)	0.66946 (14)	0.04489 (14)	0.0217 (3)	
H17A	−0.0427	0.6118	0.0237	0.026*	
H17B	0.0442	0.6637	−0.0347	0.026*	

### Atomic displacement parameters (Å^2^)

**Table:**

	*U*^11^	*U*^22^	*U*^33^	*U*^12^	*U*^13^	*U*^23^
O1	0.0172 (4)	0.0250 (5)	0.0207 (5)	0.0016 (4)	−0.0013 (4)	−0.0017 (4)
O2	0.0204 (5)	0.0185 (5)	0.0223 (5)	−0.0015 (4)	−0.0023 (4)	−0.0026 (4)
N1	0.0150 (5)	0.0125 (5)	0.0151 (5)	−0.0002 (4)	0.0036 (4)	0.0007 (4)
N2	0.0198 (5)	0.0137 (5)	0.0161 (5)	−0.0013 (4)	0.0050 (4)	0.0010 (4)
N3	0.0144 (5)	0.0165 (6)	0.0307 (7)	−0.0019 (4)	−0.0017 (5)	0.0071 (5)
C1	0.0221 (7)	0.0219 (7)	0.0195 (7)	−0.0008 (5)	0.0015 (5)	0.0003 (5)
C2	0.0197 (6)	0.0134 (6)	0.0178 (6)	0.0013 (5)	0.0030 (5)	−0.0025 (5)
C3	0.0148 (6)	0.0188 (6)	0.0217 (7)	−0.0007 (5)	0.0046 (5)	−0.0014 (5)
C4	0.0183 (6)	0.0170 (6)	0.0171 (6)	0.0000 (5)	0.0066 (5)	−0.0001 (5)
C5	0.0158 (6)	0.0116 (6)	0.0147 (6)	0.0013 (4)	0.0031 (5)	−0.0023 (5)
C6	0.0153 (6)	0.0142 (6)	0.0189 (6)	0.0003 (5)	0.0061 (5)	−0.0016 (5)
C7	0.0223 (6)	0.0141 (6)	0.0162 (6)	0.0009 (5)	0.0071 (5)	−0.0008 (5)
C8	0.0127 (6)	0.0154 (6)	0.0171 (6)	−0.0001 (4)	0.0057 (5)	−0.0013 (5)
C9	0.0153 (6)	0.0147 (6)	0.0160 (6)	0.0009 (5)	0.0052 (5)	0.0005 (5)
C10	0.0199 (6)	0.0150 (6)	0.0149 (6)	0.0002 (5)	0.0046 (5)	0.0014 (5)
C11	0.0141 (6)	0.0182 (6)	0.0157 (6)	−0.0001 (5)	0.0062 (5)	0.0017 (5)
C12	0.0179 (6)	0.0157 (6)	0.0202 (6)	0.0011 (5)	0.0063 (5)	0.0011 (5)
C13	0.0178 (6)	0.0198 (7)	0.0204 (7)	0.0045 (5)	0.0045 (5)	0.0046 (5)
C14	0.0132 (6)	0.0238 (7)	0.0143 (6)	0.0002 (5)	0.0026 (5)	0.0015 (5)
C15	0.0160 (6)	0.0166 (6)	0.0183 (6)	−0.0010 (5)	0.0057 (5)	−0.0003 (5)
C16	0.0141 (6)	0.0171 (6)	0.0181 (6)	0.0016 (5)	0.0035 (5)	0.0025 (5)
C17	0.0180 (6)	0.0242 (7)	0.0190 (6)	−0.0007 (5)	0.0013 (5)	−0.0026 (5)

### Geometric parameters (Å, º)

**Table:**

O2—C15	1.3787 (16)	C4—H4	0.9500
O2—C17	1.4347 (15)	C5—C6	1.3872 (18)
O1—C14	1.3856 (15)	C6—C7	1.3911 (17)
O1—C17	1.4354 (17)	C6—H6	0.9500
N1—C8	1.3649 (16)	C7—H7	0.9500
N1—N2	1.3810 (15)	C8—C9	1.3925 (17)
N1—C5	1.4258 (15)	C9—C10	1.4105 (17)
N2—C10	1.3183 (16)	C9—C11	1.4719 (17)
N3—C8	1.3621 (17)	C10—H10	0.9500
N3—H1N	0.883 (9)	C11—C12	1.4016 (18)
N3—H2N	0.875 (9)	C11—C16	1.4134 (18)
C1—C2	1.5091 (17)	C12—C13	1.4032 (17)
C1—H1A	0.9800	C12—H12	0.9500
C1—H1B	0.9800	C13—C14	1.372 (2)
C1—H1C	0.9800	C13—H13	0.9500
C2—C7	1.3940 (19)	C14—C15	1.3830 (18)
C2—C3	1.3946 (19)	C15—C16	1.3771 (17)
C3—C4	1.3898 (17)	C16—H16	0.9500
C3—H3	0.9500	C17—H17A	0.9900
C4—C5	1.3921 (18)	C17—H17B	0.9900
			
C15—O2—C17	104.43 (10)	N3—C8—N1	122.88 (11)
C14—O1—C17	104.05 (9)	N3—C8—C9	130.31 (12)
C8—N1—N2	111.85 (10)	N1—C8—C9	106.77 (11)
C8—N1—C5	129.22 (11)	C8—C9—C10	103.96 (11)
N2—N1—C5	118.74 (10)	C8—C9—C11	129.77 (12)
C10—N2—N1	104.01 (10)	C10—C9—C11	126.12 (11)
C8—N3—H1N	122.2 (11)	N2—C10—C9	113.40 (11)
C8—N3—H2N	117.3 (11)	N2—C10—H10	123.3
H1N—N3—H2N	119.0 (16)	C9—C10—H10	123.3
C2—C1—H1A	109.5	C12—C11—C16	119.12 (12)
C2—C1—H1B	109.5	C12—C11—C9	119.26 (12)
H1A—C1—H1B	109.5	C16—C11—C9	121.47 (11)
C2—C1—H1C	109.5	C11—C12—C13	122.76 (12)
H1A—C1—H1C	109.5	C11—C12—H12	118.6
H1B—C1—H1C	109.5	C13—C12—H12	118.6
C7—C2—C3	118.16 (12)	C14—C13—C12	116.48 (12)
C7—C2—C1	120.64 (12)	C14—C13—H13	121.8
C3—C2—C1	121.20 (12)	C12—C13—H13	121.8
C4—C3—C2	121.06 (12)	C13—C14—C15	121.64 (12)
C4—C3—H3	119.5	C13—C14—O1	128.63 (12)
C2—C3—H3	119.5	C15—C14—O1	109.69 (12)
C3—C4—C5	119.71 (12)	C16—C15—O2	127.53 (12)
C3—C4—H4	120.1	C16—C15—C14	122.83 (12)
C5—C4—H4	120.1	O2—C15—C14	109.63 (11)
C6—C5—C4	120.25 (11)	C15—C16—C11	117.16 (11)
C6—C5—N1	120.59 (11)	C15—C16—H16	121.4
C4—C5—N1	119.06 (11)	C11—C16—H16	121.4
C5—C6—C7	119.32 (11)	O2—C17—O1	107.40 (10)
C5—C6—H6	120.3	O2—C17—H17A	110.2
C7—C6—H6	120.3	O1—C17—H17A	110.2
C6—C7—C2	121.51 (12)	O2—C17—H17B	110.2
C6—C7—H7	119.2	O1—C17—H17B	110.2
C2—C7—H7	119.2	H17A—C17—H17B	108.5
			
C8—N1—N2—C10	1.15 (13)	C8—C9—C10—N2	0.51 (14)
C5—N1—N2—C10	−174.21 (10)	C11—C9—C10—N2	−175.48 (11)
C7—C2—C3—C4	0.04 (19)	C8—C9—C11—C12	158.82 (13)
C1—C2—C3—C4	−179.34 (12)	C10—C9—C11—C12	−26.24 (19)
C2—C3—C4—C5	0.3 (2)	C8—C9—C11—C16	−25.6 (2)
C3—C4—C5—C6	−0.26 (19)	C10—C9—C11—C16	149.34 (13)
C3—C4—C5—N1	176.08 (11)	C16—C11—C12—C13	−1.36 (19)
C8—N1—C5—C6	−47.97 (18)	C9—C11—C12—C13	174.33 (11)
N2—N1—C5—C6	126.47 (12)	C11—C12—C13—C14	0.57 (19)
C8—N1—C5—C4	135.70 (13)	C12—C13—C14—C15	0.53 (19)
N2—N1—C5—C4	−49.86 (16)	C12—C13—C14—O1	178.07 (12)
C4—C5—C6—C7	−0.10 (18)	C17—O1—C14—C13	168.94 (14)
N1—C5—C6—C7	−176.39 (11)	C17—O1—C14—C15	−13.28 (14)
C5—C6—C7—C2	0.44 (19)	C17—O2—C15—C16	−168.01 (13)
C3—C2—C7—C6	−0.41 (19)	C17—O2—C15—C14	13.12 (14)
C1—C2—C7—C6	178.98 (12)	C13—C14—C15—C16	−0.8 (2)
N2—N1—C8—N3	177.01 (11)	O1—C14—C15—C16	−178.81 (11)
C5—N1—C8—N3	−8.2 (2)	C13—C14—C15—O2	178.09 (12)
N2—N1—C8—C9	−0.87 (14)	O1—C14—C15—O2	0.12 (15)
C5—N1—C8—C9	173.88 (11)	O2—C15—C16—C11	−178.70 (12)
N3—C8—C9—C10	−177.44 (13)	C14—C15—C16—C11	0.03 (19)
N1—C8—C9—C10	0.23 (13)	C12—C11—C16—C15	1.02 (18)
N3—C8—C9—C11	−1.7 (2)	C9—C11—C16—C15	−174.57 (11)
N1—C8—C9—C11	176.01 (12)	C15—O2—C17—O1	−21.31 (13)
N1—N2—C10—C9	−1.00 (14)	C14—O1—C17—O2	21.29 (13)

### Hydrogen-bond geometry (Å, º)

Cg1 and Cg2 are the centroids of the C2–C7 and C11–C16 rings, respectively.

**Table:**

*D*—H···*A*	*D*—H	H···*A*	*D*···*A*	*D*—H···*A*
N3—H1*N*···N2^i^	0.88 (2)	2.16 (2)	2.9981 (16)	159 (1)
C10—H10···*Cg*1^ii^	0.95	2.97	3.6753 (14)	133
C17—H17*B*···*Cg*2^iii^	0.99	2.66	3.6334 (15)	169

Symmetry codes: (i) −*x*+1, *y*−1/2, −*z*+3/2; (ii) −*x*+1, *y*+1/2, −*z*+3/2; (iii) *x*, −*y*+1/2, *z*−3/2.
